# A Temperature Compensation Method for the Bit Parameter Recorder in High-Temperature Deep Wells Based on Thermo-Mechanical Coupling

**DOI:** 10.3390/s26061884

**Published:** 2026-03-17

**Authors:** Hengshuo Zhang, Zhenhuan Yi, Zhenbao Li, Yongyong Li, Yong Zhu

**Affiliations:** 1 National Research Center of Pumps, Jiangsu University, Zhenjiang 212013, China; 2212411027@stmail.ujs.edu.cn; 2 College of Petroleum, China University of Petroleum-Beijing at Karamay, Karamay 834000, China; y2024216880@st.cupk.edu.cn; 3 Institute of Engineering Technology, PetroChina Western Drilling Engineering Co., Ltd., Karamay 834000, China; lzbgcy@cnpc.com.cn (Z.L.); leey3515@cnpc.com.cn (Y.L.)

**Keywords:** high-temperature deep wells, measurement while drilling, thermo-mechanical coupling, temperature compensation

## Abstract

Measurement While Drilling (MWD) tools are widely employed in deep and ultra-deep well drilling. In the high-temperature and high-pressure (HTHP) environments characteristic of these wells, structural deformation induced by thermal expansion interferes with the bit parameter recorder’s sensor readings, thereby degrading the measurement accuracy of weight on bit (WOB) and working torque (WT). To address this issue, this paper proposes a temperature compensation method based on thermo-mechanical coupling simulation. This method systematically establishes the quantitative relationships between multiple loads—including WT, WOB, temperature, and make-up torque—and the strain at critical locations of the bit parameter recorder through finite element analysis (FEA). Furthermore, surface calibration experiments have verified a strong linear correlation between the strain gauge voltage signals and the simulated strain. Building upon this foundation, an inversion-based compensation algorithm is developed. This algorithm effectively isolates the interference caused by thermally induced deformation and inversely deduces the true WOB and torque values by utilizing downhole-measured sensor voltage and temperature data. The research results demonstrate that the proposed temperature compensation method significantly improves the measurement accuracy of the bit parameter recorder under harsh, high-temperature operating conditions. The relative errors for both WOB and torque measurements are controlled to within 5%, providing a reliable solution for precise parameter measurement in high-temperature deep wells.

## 1. Introduction

**Figure 1:**
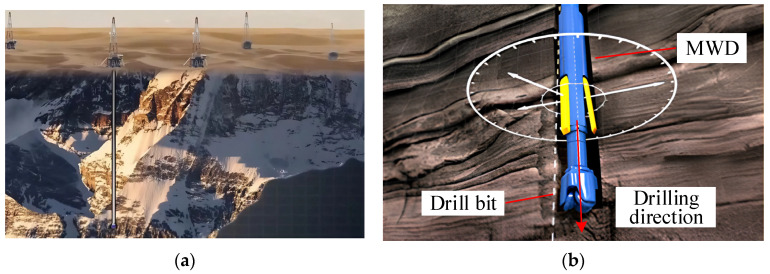
Schematic diagram of deep well drilling technology (**a**) Deep well drilling (**b**) Automatic vertical drilling system.

Energy exploration is progressively targeting deeper hydrocarbon reservoirs and unconventional formations. Consequently, Measurement While Drilling (MWD) systems now operate in downhole environments with increasingly severe temperature and pressure conditions [1]. In such high-temperature settings, conventional bit-parameter recorders face significant challenges in maintaining measurement reliability. This limitation constitutes a critical obstacle to the efficient development of deep oil and gas resources (Figure 1a). Notably, current scientific drilling depths have surpassed 10,000 m, further driven by the development of geothermal resources like hot dry rock. As a result, downhole ambient temperatures frequently exceed the critical threshold of 200 °C. Under this extreme thermal loading, material thermal expansion induces structural deformation in MWD tools (Figure 1b). This deformation triggers systematic drift in sensor signals. Consequently, the measured values for critical parameters, such as Weight on Bit (WOB) and Torque, deviate severely from their true mechanical load values [2].

Within the parameters monitored by MWD, accurate measurement of Weight on Bit (WOB) and Working Torque (WT) is critical. These parameters are essential for precise well trajectory control, improved Rate of Penetration (ROP), and enhanced drilling safety [3,4]. However, a major challenge persists in existing near-bit MWD tools that rely on strain gauges. Temperature fluctuations cause material thermal expansion and alter the internal thermal-stress distribution of the tool structure. This effect leads to significant temperature-induced drift in the strain gauge output signals [5]. To mitigate the degradation in sensor accuracy caused by high temperatures, current research primarily follows two pathways: hardware compensation and software compensation [6,7]. Hardware compensation aims to improve the sensor’s inherent high-temperature performance by advancing its design, materials, or fabrication processes.

In terms of sensor structure, research has focused on developing novel high-temperature thin-film strain gauges. For example, Zhao et al. [8] systematically reviewed the material systems, fabrication processes, and performance optimization strategies for these gauges. Their work indicates that precious metals and ceramic composites are viable solutions for extreme-temperature applications. Separately, Wang et al. [9] targeted ultra-high-temperature environments exceeding 800 °C. They reviewed various strain gauge technologies, including metal alloys, ceramics, and single-crystal semiconductors, and analyzed their high-temperature stability and failure mechanisms.

Unlike hardware compensation, software compensation methods leave the sensor hardware unchanged [10,11,12]. Instead, they apply mathematical models or intelligent algorithms to post-process the sensor’s raw output signals, thereby correcting temperature-induced errors. The core of this approach is to use ambient-temperature data from dedicated temperature sensors to compensate for the readings of target physical quantities, such as strain or pressure [13,14]. Based on algorithmic principles, software compensation can be divided into parametric model-based methods and data-driven methods. Parametric model-based methods typically require establishing an explicit mathematical relationship between temperature and sensor output. Interpolation-based temperature compensation method for downhole high-temperature pressure sensors. Their method performs interpolation within a two-dimensional grid of temperature-pressure calibration data, enabling rapid and accurate calculation of compensated pressure values. This approach offers high computational efficiency and is well-suited for implementation in embedded systems.

For temperature drift problems exhibiting severe nonlinearity or complex underlying mechanisms, data-driven, non-parametric machine-learning methods demonstrate significant advantages [15]. These methods can automatically learn the complex patterns of temperature influence from large amounts of calibration data. Wang et al. [16] proposed a sensor temperature compensation strategy employing a Firefly Algorithm-optimized Backpropagation (BP) neural network, which uses the optimization algorithm to enhance the neural network’s performance, resulting in a compensation model with higher accuracy and stronger robustness. Wang et al. [17] combined the artificial lemming algorithm with the eXtreme Gradient Boosting model to develop a hybrid temperature compensation algorithm for pressure scanners. This ALA-XGBoost model achieved excellent performance in experiments, robustly validating the feasibility and effectiveness of the ensemble learning model for temperature compensation in complex equipment applications [18].

Although significant progress has been made in existing research, current temperature-compensation methods still have notable limitations for strain-gauge-based mechanical-parameter sensors. On one hand, most studies have focused on temperature compensation for nuclear and electrical logging tools, while research specifically addressing temperature drift in strain measurements remains relatively scarce. On the other hand, existing thermo-mechanical coupling models often simplify the heat transfer boundary conditions between the tool and the downhole environment, failing to fully account for the dynamic characteristics of drilling fluid convective heat transfer, thereby creating a gap between model prediction accuracy and practical requirements.

In summary, there is currently no temperature-compensation method specifically designed for the bit parameter recorder that accounts for complex thermo-mechanical coupling effects. The innovative contributions of this paper are as follows:

(1) An integrated “simulation-calibration-inversion” temperature compensation framework was constructed. Through thermo-mechanical coupling simulation, this framework systematically establishes a quantitative mapping relationship between temperature, multiple mechanical loads (Weight on Bit, Working Torque, Make-up Torque), and the strain at critical sensor locations, overcoming the challenge of directly modeling and decoupling the complex downhole thermo-mechanical coupling environment.
(2) A high-accuracy temperature compensation algorithm with strong engineering practicality was developed. Based on simulation data and surface calibration experiments, a bivariate quadratic polynomial regression model was established, which takes measured temperature and voltage as inputs and outputs true Weight on Bit and Torque. This algorithm does not rely on hardware modifications and offers high computational efficiency, significantly improving measurement accuracy at high temperatures and providing a viable solution for field applications.

The remainder of this paper is organized as follows: Section 2 details the working principle of the bit parameter recorder and analyzes how temperature affects its measurements. Section 3 presents the developed thermo-mechanical coupling simulation model and discusses key findings from the analysis. Section 4 describes the design and execution of calibration experiments for torque and WOB. This section also explains how experimental and simulation data are integrated to build and validate the compensation algorithm. Finally, Section 5 summarizes the main conclusions drawn from this research.

## 2. Bit Parameter Recorder and Temperature Influence Mechanism

### 2.1. Working Principle of the Bit Parameter Recorder

The bit parameter recorder, serving as a critical near-bit MWD tool, is primarily designed to monitor and record key mechanical parameters, such as WOB and WT, during drilling. Upon completion of drilling, the recorded parameters can be retrieved by connecting the tool to a data analysis system, enabling subsequent bit dynamics analysis. This instrument operates on the strain-gauge measurement principle, achieving precise acquisition of mechanical parameters by detecting minute deformations of the drill string under complex drilling loads [19].

**Figure 2:**
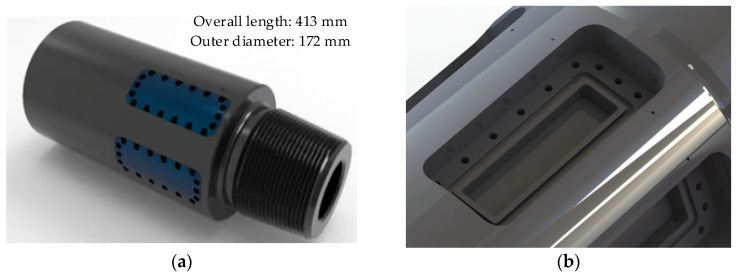
The drill bit parameter recorder. (**a**) 3D model of drill bit parameter recorder; (**b**) Mounting groove for the strain gauge sensor.

The main body of the parameter recorder features a monolithic design with three sensor mounting grooves near the pin connection. Figure 2 shows the 3D model and the mounting groove for the strain gauge sensor. Each groove integrates three core components: a sensing unit, a signal processing unit, and a data storage unit. The sensing unit employs multiple sets of strain gauges to directly detect changes in the drill string’s mechanical state. Subsequently, the signal processing unit digitizes the weak strain signals through amplification and filtering. Finally, the data storage unit preserves the measurement data reliably under HTHP conditions, forming the basis for subsequent drill string dynamics analysis.

To ensure durability in harsh downhole environments, the parameter recorder is fabricated entirely from the 15-15 HS MAX nitrogen-strengthened austenitic stainless steel. This material selection guarantees long-term operational stability [20]. During measurement, mechanical deformation of the drill string is first converted into a change in electrical resistance by the bonded strain gauges. Subsequently, a Wheatstone bridge circuit transforms this resistance variation into a measurable voltage signal. A key characteristic of this system is the proportional relationship between the output voltage and the induced strain. This proportionality provides the fundamental mathematical link that maps the electrical output to the magnitude of the applied mechanical load.

The operational workflow of the recorder adheres to a strict sequential control: following power-on initialization, the system performs self-test and parameter calibration, then enters a continuous data-acquisition mode. All measured data are timestamped with precise timing, thereby providing data support for subsequent drilling process optimization.

#### 2.1.1. Measurement Principle of the Torque Sensor

The torque sensor in this recorder utilizes the piezoresistive effect for measurement. This effect describes how the electrical resistance of a bonded strain gauge changes when it undergoes mechanical deformation. The fundamental relationship between this resistance change and the mechanical strain is expressed by Equation (1), where Δ*R*/*R* represents the relative change in resistance, *K*_0_ is the material-specific gauge factor, and *ε* denotes the induced strain [21]

(1) $$\frac{\Delta R}{R}=K_{0}\varepsilon$$

**Figure 3:**
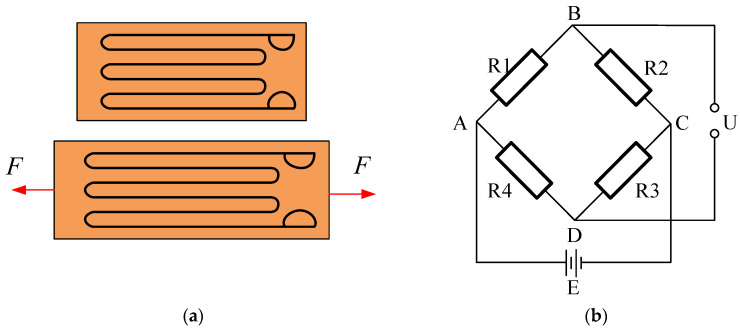
Strain gauge torque sensor (**a**) Strain gauge under tension (**b**) Strain gauge full-bridge circuit.

Figure 3a illustrates this core relationship. For the detection circuit, a Wheatstone full-bridge configuration is employed, shown in Figure 3b. The bridge integrates four strain gauges. Two are mounted at a 45° orientation relative to the tool axis, while the other two are at 135°. This specific arrangement aligns the gauges with the directions of maximum positive and negative shear strain. During drilling, the application of WT generates a proportional shear-stress field on the groove surface. Consequently, the 45° gauges are subjected to tensile strain, and the 135° gauges to compressive strain. This differential deformation unbalances the bridge, producing a net output voltage that is linearly related to the shear strain [22].

The four strain gauges in the Wheatstone full-bridge circuit have identical resistance values, i.e., *R*_1_ = *R*_2_ = *R*_3_ = *R*_4_. With E representing the voltage of the power source, the output voltage *U*_BD_ of the circuit is:

(2) $$U_{\mathrm{BD}}=U_{\mathrm{AB}}-U_{\mathrm{AD}}=\frac{R_{1}}{R_{1}+R_{2}}E-\frac{R_{3}}{R_{3}+R_{4}}E=\frac{R_{1}R_{4}-R_{2}R_{3}}{\left(R_{1}+R_{2})(R_{3}+R_{4}\right)}E$$

In the full-bridge configuration, since *R*_1_*R*_4_ = *R*_2_*R*_3_, the bridge is in a balanced state, resulting in a voltage output *U*_BD_ = 0. Given that *R*_1_ = *R*_2_ = *R*_3_ = *R*_4_ = *R*, and that the opposing arms of the bridge experience forces of the same nature, when the strain gauges deform under load, their resistances change as Δ*R*_1_ = Δ*R*_4_ = Δ*R* and Δ*R*_2_ = Δ*R*_3_ = −Δ*R*. The output voltage under this condition becomes:

(3) $${V_{\mathrm{out}}}^{\prime }=\frac{{\left(R+\Delta R\right)}^{2}-{\left(R-\Delta R\right)}^{2}}{2R\cdot 2R}V_{\mathrm{ex}}=\frac{\Delta R}{R}V_{\mathrm{ex}}=K_{0}\varepsilon V_{\mathrm{ex}}$$

The theoretical derivation of torque measurement centers on the shear stress–strain relationship in mechanics. Specifically, applying a torque *T* to the drill string induces a shear stress field on the surface where the sensor is mounted. The maximum shear stress *τ*_max_ at this surface relates to the applied torque by Equation (4). In this expression, *r* denotes the radius of the critical cross-section, and *J* is its polar moment of inertia.

(4) $$\tau_{\max}=\frac{Tr}{J}$$

Within the material’s elastic limit, shear stress *τ* and shear strain *γ* are linearly proportional, as defined by Hooke’s law: Equation (5). The constant of proportionality G is the material’s shear modulus.

(5) τ=Gγ

The output from the Wheatstone bridge circuit provides the link to this mechanical strain. The bridge’s output voltage *V*_out_ relates to the shear strain γ and the excitation voltage *V*_ex_ as shown in Equation (6).

(6) $$V_{\mathrm{out}}=\frac{K\gamma V_{\mathrm{ex}}}{4}$$

Integrating the relationships from Equations (4)–(6) yields the final model. This integration establishes a direct, usable relationship between the mechanical torque *T* and the measured electrical output *V*_out_:

(7) $$T\;=\;\frac{2GJV_{\mathrm{out}}}{KrV_{\mathrm{ex}}}$$

#### 2.1.2. Measurement Principle of the WOB Sensor

The WOB sensor operates on the principle of axial strain detection. Its design similarly incorporates a Wheatstone full-bridge circuit. Within the mounting groove, active strain gauges are aligned with the drill string axis. This orientation allows them to directly sense axial deformation resulting from the applied WOB [23]. Under WOB loading, the recorder experiences axial compressive strain. This strain alters the resistance of the active gauges, which unbalances the bridge circuit. Consequently, the bridge generates an output voltage signal that is proportional to the induced strain [24].

The underlying theory relates axial stress to strain. When WOB is applied, the resulting axial stress *σ* is related to the force *F*_w_ by Equation (8), where *A* represents the effective cross-sectional area of the measurement region.

(8) $$\sigma =\frac{F_{w}}{A}$$

Following Hooke’s law for linear elasticity, the axial strain *ε* is proportional to the stress *σ*, as expressed in Equation (9). The constant *E* in this relationship is the material’s Young’s modulus.

(9) σ=Eε

where *E* is the material’s Young’s modulus. The relationship between the resistance change rate of the strain gauge and the axial strain is determined by the gauge factor *K*. For a half-bridge circuit configuration, the relationship between the output voltage *V*_out_ and the strain *ε* can be expressed as:

(10) $$V_{\mathrm{out}}\;=\;\frac{K\varepsilon V_{\mathrm{ex}}}{2}$$

Combining the mechanical and electrical relationships from Equations (8)–(10) yields the definitive input-output model for the sensor. This derivation produces the quantitative expression seen in Equation (11), which directly relates the measured output voltage *V*_out_ to the applied Weight on Bit *F*_w_.

(11) $$F_{w}=\frac{2AEV_{\mathrm{out}}}{KV_{\mathrm{ex}}}$$

### 2.2. Analysis of Temperature Influence Mechanism

The influence of temperature on the bit parameter recorder and near-bit MWD tools is a complex process involving multi-physics field coupling [25,26]. The underlying mechanism can be systematically analyzed from four dimensions: material, structure, measurement circuit, and downhole environment. The combined effect of these factors leads to a significant degradation in sensor measurement accuracy under high-temperature conditions.

At the material level, rising temperatures cause two critical changes: thermal expansion and reduced mechanical stiffness. For an isotropic material, the induced thermal strain tensor is given by Equation (12). In this expression, α represents the coefficient of linear thermal expansion, Δ*T* is the temperature change, and *δ*_ij_ is the Kronecker delta, defined as *δ*_ij_ = 1 if i = j and *δ*_ij_ = 0 if i ≠ j.

(12) $$\varepsilon_{\mathrm{ij}}^{\mathrm{th}}=\alpha \Delta T\delta_{\mathrm{ij}}$$

where α is the material’s coefficient of linear thermal expansion, ΔT is the temperature change, and *δ*_ij_ is the Kronecker delta. For the 15-15 HS MAX nitrogen-strengthened austenitic stainless steel used in the bit parameter recorder, α exhibits a nonlinear variation with temperature:

(13) $$\alpha (T)=\alpha_{0}+\alpha_{1}T+\alpha_{2}T^{2}$$

This thermal expansion effect translates into thermal stress under structural constraints, according to the Duhamel-Neumann law:

(14) $$\sigma_{\mathrm{ij}}=C_{\mathrm{ijkl}}(\varepsilon_{\mathrm{kl}}-\varepsilon_{\mathrm{kl}}^{\mathrm{th}})$$

When constrained by the structure, this thermal strain generates internal stress. The Duhamel-Neumann law describes this thermoelastic coupling, as shown in Equation (14), where *C*_ijkl_ is the fourth-order elastic stiffness tensor.

(15) $$E(T)=E_{0}\left[1-\beta_{E}(T-T_{0})\right]$$

where *E* is the temperature coefficient of Young’s modulus, and *T*_0_ is the initial temperature. This temperature-dependent stiffness results in a greater strain response under identical mechanical loads at elevated temperatures.

The strain gauge system itself is inherently temperature-sensitive. The total resistance change in a gauge combines mechanical and thermal contributions, as expressed in Equation (16).

(16) $$\frac{\Delta R}{R}=K\varepsilon +\left[\beta_{g}+K(\alpha_{s}-\alpha_{g})\right]\Delta T$$

where *K* is the gauge factor, *β*_g_ is the temperature coefficient of resistivity for the strain gauge wire, *α*_s_ is the coefficient of thermal expansion of the substrate material, and *α*_g_ is the coefficient of thermal expansion of the strain gauge. In a Wheatstone bridge configuration, the spurious output caused by temperature, $V_{\mathrm{out}}^{\mathrm{th}}$, is quantified by Equation (17), where *V*_cc_ is the bridge excitation voltage, and *β*_gi_, *α*_gi_ and Δ*T*_i_ are the resistivity coefficient, expansion coefficient, and temperature rise for the i-th gauge, respectively. As shown, the thermal output has a complex relationship with Δ*T*, influenced by bridge configuration, material properties, and temperature distribution.

(17) $$V_{\mathrm{out}}^{\mathrm{th}}=\frac{V_{\mathrm{cc}}}{4}\sum_{i=1}^{4}\left[\beta_{\mathrm{gi}}+K(\alpha_{s}-\alpha_{\mathrm{gi}})\right]\Delta T_{i}$$

This temperature effect causes significant output drift in the strain gauges even in the absence of external loads. The heat-exchange process in the downhole environment further complicates the temperature influence [27]. The convective heat transfer between the drilling fluid and the outer wall of the parameter recorder is described by Newton’s law of cooling:

(18) $$q=h(T_{s}-T_{f})$$

where *h* is the convective heat transfer coefficient, and *T*_s_ and *T*_f_ are the wall surface and drilling fluid temperatures, respectively. The heat transfer coefficient *h* is closely related to the flow state of the drilling fluid:

(19) $$h=\frac{Nu\cdot k_{f}}{D_{h}}$$

(20) $$Nu=\frac{(f/8)(Re-1000)Pr}{1+12.7\sqrt{f/8}\left(Pr^{2/3}-1\right)}$$

The coefficient *h* depends on the fluid flow state, as shown in Equation (19), involving the Nusselt number (*Nu*), fluid thermal conductivity (*k*_f_), and hydraulic diameter (*D*_h_). For annular turbulent flow, *Nu* is given by the Gnielinski correlation in Equation (20), where *f* is the Darcy friction factor, *Re* is the Reynolds number, and *Pr* is the Prandtl number [28]. This dynamic heat exchange causes continuous surface temperature fluctuations, introducing additional noise.

In summary, the mechanisms of temperature’s influence on the bit parameter recorder can be categorized as follows: First, material thermal expansion alters the structural geometry, directly modifying the strain distribution. Second, the temperature dependence of material properties changes the mechanical response characteristics. Third, the inherent temperature effects of the strain gauges themselves introduce measurement bias. Fourth, the instability of the downhole thermal environment introduces dynamic disturbances. The coupling of these factors makes temperature one of the critical factors affecting MWD accuracy, necessitating precise thermo-mechanical coupling analysis and effective temperature compensation methods for resolution.

## 3. Thermo-Mechanical Coupling Simulation Model

### 3.1. Modeling Methodology for the Thermo-Mechanical Coupling Model

Accurately simulating the parameter recorder under combined high-temperature and multi-load conditions requires a thermo-mechanical coupling approach. This methodology simulates the stress and strain states during drilling and their effect on the embedded strain gauge sensors. The simulation employs a sequential coupling strategy. This method solves the temperature and stress fields separately, in consecutive steps. It maintains computational accuracy while enhancing efficiency [28]. By decomposing the multi-physics problem into simpler, sequential processes coupled through data transfer [29], this strategy is particularly effective for drilling analysis. In this context, temperature field changes are relatively slow, and the mechanical response can be treated as quasi-static.

The simulation workflow follows three primary stages: pre-processing, solution, and post-processing. In the pre-processing stage, a precise 3D geometric model of the recorder is created. Features with negligible thermal-structural influence, such as certain threaded holes, are reasonably simplified [30]. Next, temperature-dependent material properties are defined. These include Young’s modulus, Poisson’s ratio, the coefficient of thermal expansion, thermal conductivity, and specific heat capacity. Finally, boundary conditions are applied. This setup accounts for convective heat transfer between the drill string surface and the drilling fluid, using a heat transfer coefficient model adjusted for the Reynolds number [31].

The solution phase implements the sequential coupling strategy. This begins with a steady-state thermal analysis of the full recorder model to solve the 3D heat conduction equation, yielding the temperature field distribution. The governing equation for this analysis is Equation (21), where the material thermal conductivity is *k*, T represents the temperature field, and Q accounts for any internal heat source.

(21) ∇⋅(k∇T)+Q=0

Once a converged temperature field is obtained, the nodal temperatures are mapped as thermal body loads into the structural analysis module. This subsequent analysis, grounded in thermoelastic theory, solves the stress equilibrium equation that incorporates thermal effects, given by Equation (22). Here, σ denotes the stress tensor, and F is the body force vector:

(22) ∇⋅σ+F=0

The total strain ε in the material is the sum of mechanical strain *ε*_m_ and thermal strain *ε*_th_, as defined in Equation (23). The thermal strain component is calculated using the material’s coefficient of thermal expansion α and the temperature change ΔT at each point:

(23) $$\varepsilon_{\mathrm{total}}=\varepsilon_{\mathrm{mech}}+\varepsilon_{\mathrm{th}}=\varepsilon_{\mathrm{mech}}+\alpha \Delta T$$

**Figure 4:**
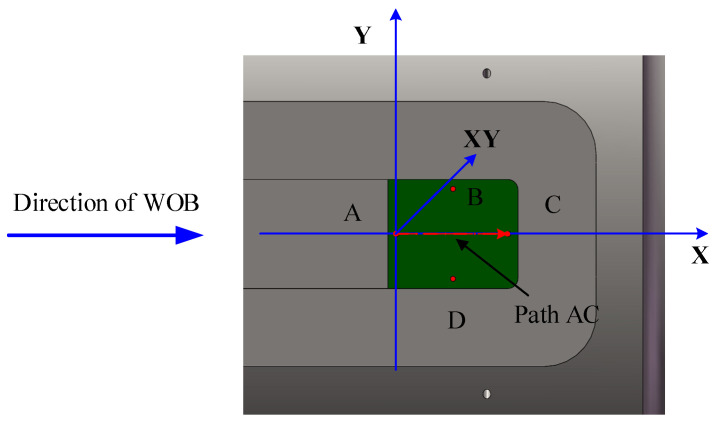
Schematic diagram of the deformation probe location.

To acquire stress–strain data in the region where the strain gauges are mounted, four deformation probes, A, B, C, and D, are positioned within this area, as shown in Figure 4. To avoid edge stress concentrations, they are located 2 mm inward from the midpoints of the four sides of the strain gauge mounting region. This multi-probe design provides measurement redundancy, ensuring system reliability even if data from a single probe fails. The strain data from the four probes can be combined to separate the axial and shear strain components. By establishing two orthogonal measurement paths, A-C and B-D, corresponding to the primary sensitive directions for WOB and torque, respectively, effective decoupling of WOB and torque is achieved.

The selection of key strain components is based on the drill string’s mechanical behavior and the specific measurement objectives. After comprehensive analysis, the X-direction elastic strain and the XY-direction shear strain were identified as the target variables.

The X-direction elastic strain serves as the primary signal for WOB measurement. Within the elastic regime, axial stress and strain obey Hooke’s law. Consequently, the applied WOB (Fw) relates to the average axial stress (${\bar{\sigma }}_{x})$ as shown in Equation (24), which incorporates the cross-sectional area A and the average axial strain ${\bar{\varepsilon }}_{x}$.

(24) $$WOB=A{\bar{\sigma }}_{x}=AE{\bar{\varepsilon }}_{x}$$

For torque measurement, the XY-direction shear strain provides the essential indicator. Torsion theory governs this relationship. The torque (*τ*) is linked to the induced surface shear strain (*γ_xy_*) by Equation (25), where *G* is the shear modulus, *J* is the polar moment of inertia, and *r* is the radius of the critical cross-section.

(25) $$\tau =\frac{GJ}{r}\gamma_{\mathrm{xy}}$$

The XY shear strain directly quantifies torsional deformation induced by torque, whereas the X-direction strain captures axial deformation from WOB. Within the strain tensor, these two components are orthogonal and mechanically independent. This inherent decoupling in their physical origins allows WOB and torque to be inversely determined from separate strain signals.

**Figure 5:**
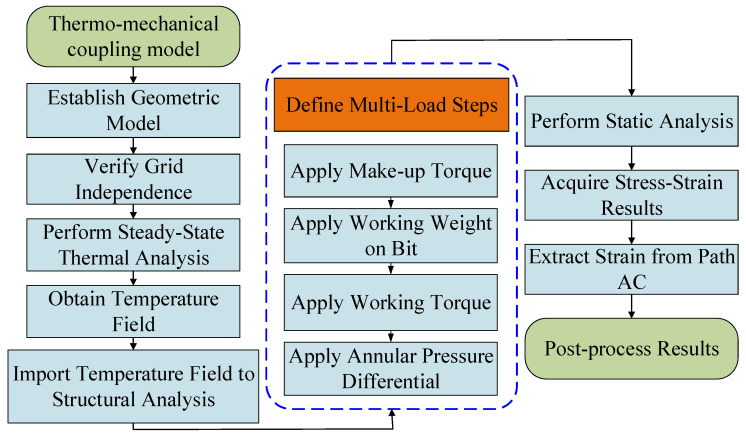
Solution procedure of the thermo-mechanical coupling model.

Through thermo-mechanical coupling simulation, the variation in these strain components with temperature and mechanical loads is quantified. This process establishes a precise mapping between the multi-field loads and the resultant strains. The resulting dataset provides the critical foundation for the temperature-compensation algorithm, enabling the accurate inversion of WOB and torque in high-temperature downhole conditions. The solution procedure of the thermo-mechanical coupling model is summarized in Figure 5.

### 3.2. Grid Independence Verification

**Figure 6:**
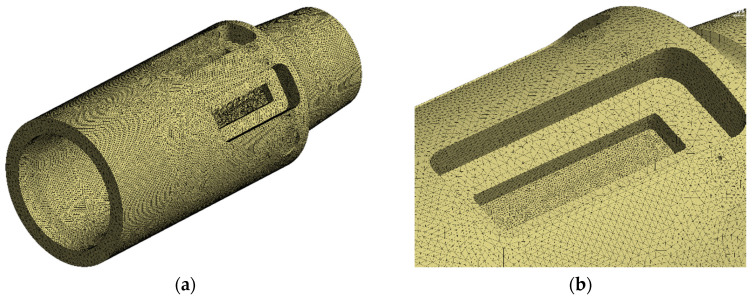
Mesh generation (**a**) Global mesh (**b**) Local mesh refinement.

During the meshing process, local refinement was applied to regions with high gradients in physical quantities. The global element size was set to 3 mm, while the element size on the surface of the sensor mounting groove was refined to 1 mm. The surfaces surrounding the groove and those of the connecting internal bore were assigned an element size of 1.5 mm; all finite element simulations in this study were conducted using ANSYS 2022R1. Figure 6 shows the global mesh and the local refinement around the sensor mounting groove.

Based on the aforementioned mesh generation strategy, five sets of computational models with varying mesh element counts were constructed by altering the mesh element size and proportionally adjusting the number of elements, as detailed in Table 1.

**Figure 7:**
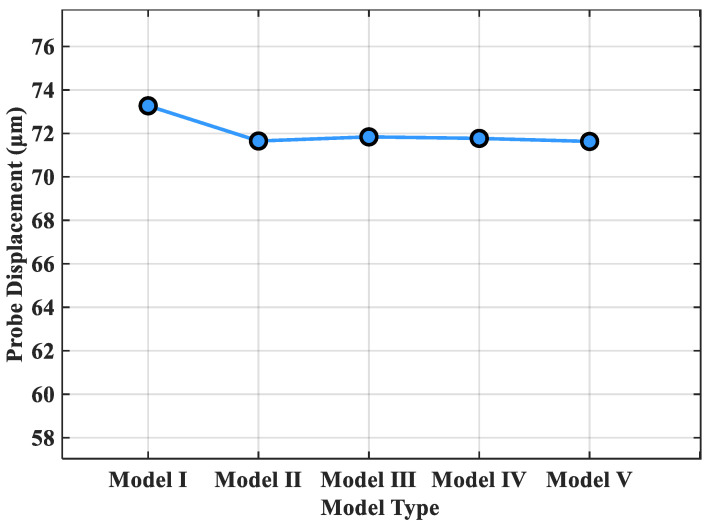
Grid independence verification.

The results of the grid independence verification are shown in Figure 7. Taking the displacement of Probe A within the sensor mounting groove as the reference metric, the displacements of Probe A under identical operating conditions were computed across the different models. From the grid independence verification results, the displacements of Probe A across the five models closely matched. Therefore, Model III was selected as the thermo-mechanical coupling computational model for this study, ensuring computational accuracy.

### 3.3. Solution of the Thermal Simulation Model

High-temperature deep-well environments feature a convective heat-transfer gap between the drill string and the borehole wall. Therefore, simulating the drilling fluid’s convective cooling effect is essential for predicting the recorder’s temperature distribution accurately.

**Figure 8:**
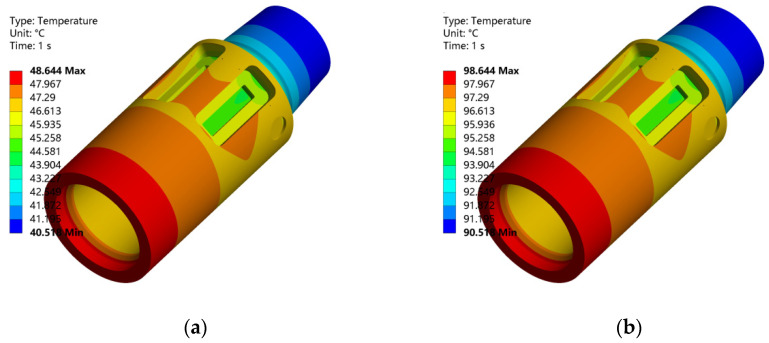

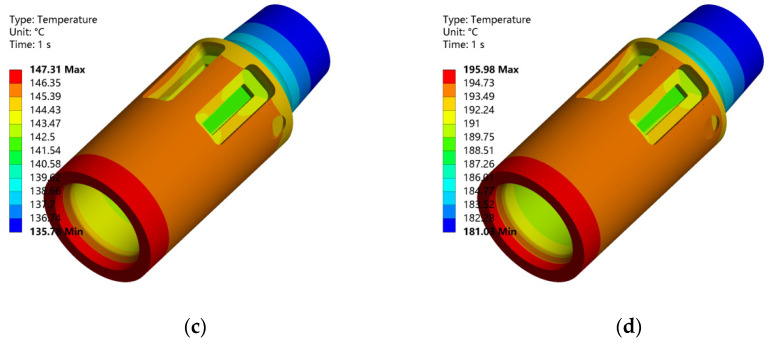
Grid Temperature distribution of the model under different ambient temperatures. (**a**) 50 °C (**b**) 100 °C (**c**) 150 °C (**d**) 200 °C.

Figure 8 presents the resulting steady-state temperature nephograms at four ambient temperatures: 50 °C, 100 °C, 150 °C, and 200 °C. These contours confirm the convective heat transfer effect. The temperature field shows a clear axial gradient. Furthermore, the unique geometry of the sensor mounting groove creates a local cooling effect. This leads to a noticeably lower surface temperature in the groove area compared to the front-end outer wall.

### 3.4. Solution of the Multi-Load Model

**Figure 9:**
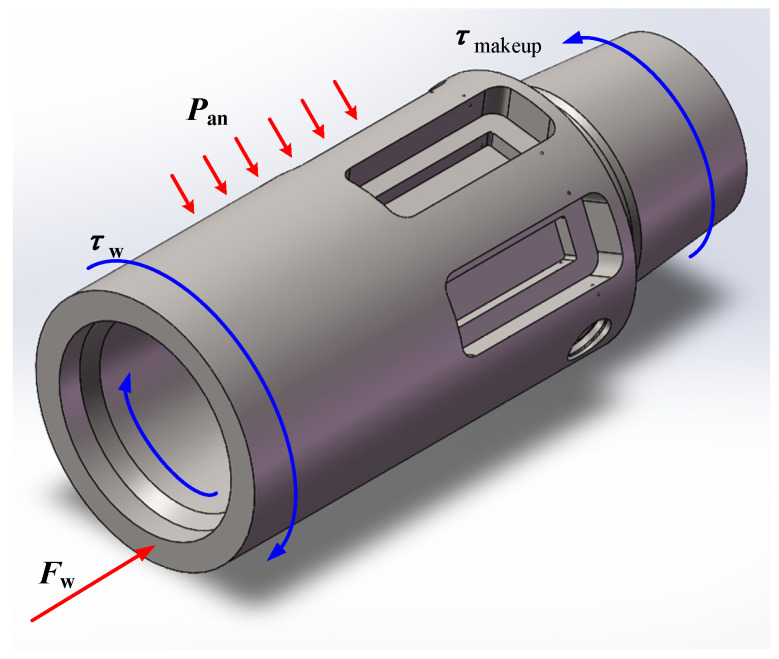
Working principles of the power integration mechanism.

The multi-load model was developed to accurately simulate the complex loading environment experienced by the bit parameter recorder during actual drilling operations. As illustrated in Figure 9, this model integrates four critical loads: the preload generated by the make-up torque, the torsional load induced by the WT, the axial compression resulting from the WOB, and the annular pressure exerted by the wellbore and drilling fluid. These loads interact synergistically in the downhole environment, collectively influencing the strain response characteristics of the recorder.

The key to multi-load simulation lies in establishing a rational sequence for applying external loads [31]. To ensure the physical authenticity of the simulation, a stepwise loading strategy was implemented based on the actual drilling process: the preload from the make-up torque due to threaded connection was first applied to simulate the initial assembly state of the tool; subsequently, with the make-up torque locked, the working weight on bit and WT were synchronously applied to simulate the actual drilling conditions for thermo-mechanical coupled analysis. This loading sequence adequately accounts for interactions between the different loads, ensuring the physical authenticity of the simulation process.

In order to comprehensively evaluate the mechanical response of the recorder under various operating conditions, a systematic parametric analysis scheme was designed [32]. The analysis variables encompass four key parameters: make-up torque (10–30 kN·m), working WOB (0–200 kN), WT (0–10 kN·m), and temperature (30–200 °C). Within the operational range, an orthogonal experimental design method was employed to construct 240 representative load combinations, ensuring the simulation results adequately cover the actual working range of the recorder [33]. Each load combination was solved independently to obtain the corresponding strain distribution data, providing a data foundation for the subsequent development of temperature-compensation algorithms.

**Figure 10:**
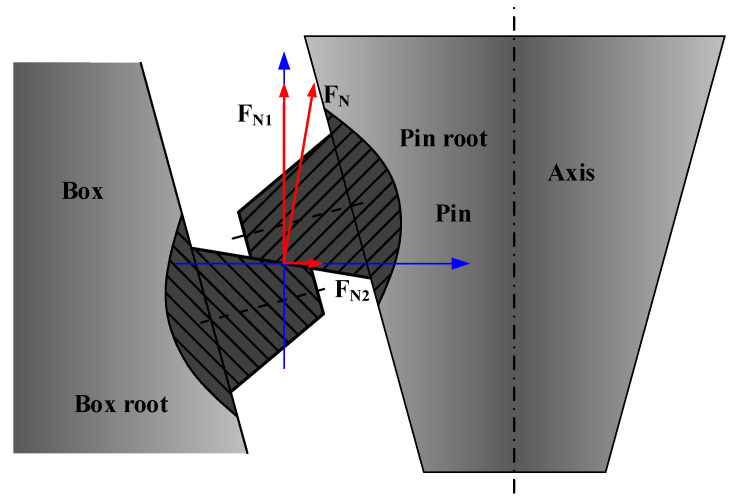
Clamping force resolution.

In the stress analysis of the bit parameter recorder, the axial preload generated by the make-up torque constitutes one of the key loads creating the initial strain field in the sensor [34]. To accurately apply this load in the simulation, the make-up torque (*τ*_makeup_) must be decomposed into the axial clamping force acting on the threaded connection surface. The mechanical conversion of the make-up torque into an axial preload in the threaded connection, specifically for the tapered threads dedicated to petroleum engineering, is illustrated in Figure 10.

The conversion from make-up torque to axial preload follows the API 4 1/2 REG thread standard. This relationship is defined by Equation (26), where *d*_2_ is the pitch diameter, *ψ* is the lead angle, and *ρ* is the friction angle (tan*ρ* = μ/cosβ, with *μ* being the coefficient of friction and *β* the thread profile angle).

(26) $$F=\frac{2\tau_{\mathrm{makeup}}}{d_{2}\cdot \tan(\psi +\rho )}$$

**Figure 11:**
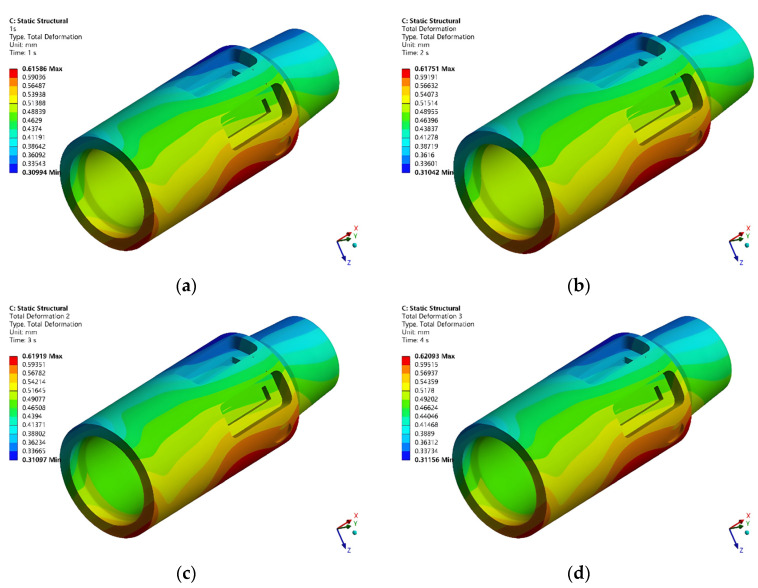
Deformation distribution of the model under different WOB: (**a**) 50 kN, (**b**) 100 kN, (**c**) 150 kN, (**d**) 200 kN.

Based on the previous analysis of the multi-load simulation sequence, after importing the temperature field distribution data into the static simulation module, a composite load simulation was performed. Figure 11 displays the overall deformation distribution of the parameter recorder under variation in WOB alone.

Following the established multi-load simulation sequence, the temperature field data were imported into the static structural module. Figure 11 specifically displays the overall deformation distribution of the parameter recorder resulting from variation in the WOB load alone.

## 4. Calibration Experiment

### 4.1. Calibration Test Bench and Data Acquisition System

**Figure 12:**
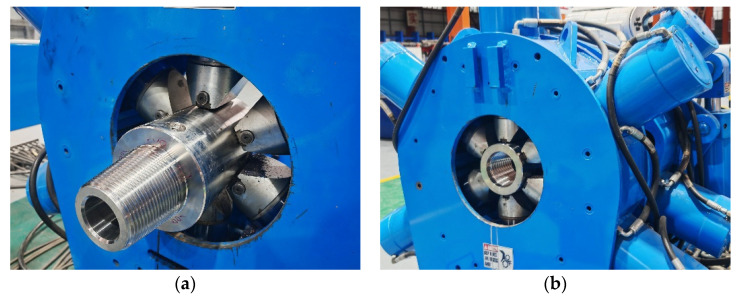

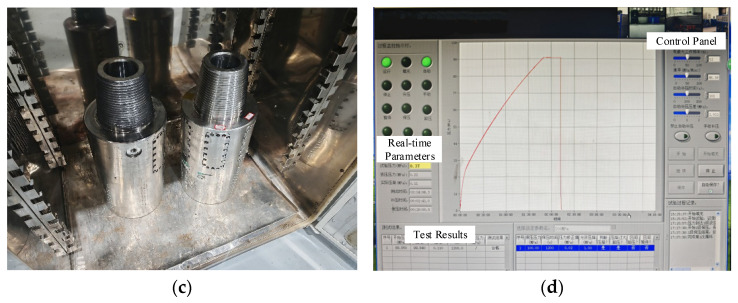
Calibration test bench and data acquisition system (**a**) Rear end of the parameter recorder (**b**) Applying working torque (**c**) Bit parameter recorder (**d**) Data acquisition system.

A surface calibration test was designed to establish an accurate load-strain relationship model. The setup (Figure 12) includes a hydraulic loading system, a standard force sensor, and a high-precision data acquisition system. The hydraulic system provides a WOB range of 0–200 kN and a torque range of 0–10 kN·m, with an accuracy of ±0.5% FS [35].

### 4.2. Analysis of Experimental Results

To ensure calibration fidelity and reduce the impact of random noise and measurement error, each torque load level was applied three times. The average of these three measurements was then calculated and recorded as the final voltage output for that load. All tests were conducted at a stable room temperature to eliminate thermal interference. The applied torque ranged from 0 to 10 kN·m, covering the sensor’s full operational scale.

The measurement principle detailed earlier confirms a strong correlation between voltage output and XY-direction shear strain. To mirror the experimental conditions, a corresponding thermo-mechanical coupled simulation was conducted. In this simulation, the ambient temperature was fixed at 25 °C, and a pure torque load was applied to the model. The resulting XY-direction shear microstrain along path AC was extracted. These combined experimental and simulation results are presented in Table 2.

Similarly, under the WOB-only condition, a WOB ranging from 0 to 200 kN was applied incrementally, covering the full measurement range of the sensor. The voltage output from the WOB channel was recorded after stabilization at each load point. A corresponding thermo-mechanical coupled simulation was designed for this WOB-only condition, during which the X-direction normal elastic microstrain *με*_x_ along the path AC was recorded. The WOB fitting experimental results are presented in Table 3.

Based on the load-voltage correspondence data obtained from the aforementioned experiments, linear regression fitting was performed using the least-squares method. Since the strain gauge sensor circuit is configured as a Wheatstone bridge and exhibits a favorable linear relationship between the voltage output and the applied load within the elastic range, a univariate linear model was adopted to derive the calibration formulas. The calibration model for the torque channel is expressed as:

(27) $$T_{\mathrm{work}}=K_{\mathrm{tor}}\cdot V_{\mathrm{tor}}+B_{\mathrm{tor}}$$

The calibration model for the torque channel is given by Equation (27), where *K*_tor_ represents the sensitivity coefficient, and *B*_tor_ denotes the zero drift of the torque channel. Similarly, the calibration model for the WOB channel is expressed as:

(28) $$F_{W}=K_{\mathrm{wob}}\cdot V_{\mathrm{wob}}+B_{\mathrm{wob}}$$

In the equations, *K*_wob_ represents the sensitivity coefficient of the WOB channel, and *B*_wob_ denotes its zero-point drift. The linear-fitting coefficients were derived from the data presented in Table 2 and Table 3. The results are presented in Table 4. The coefficient of determination (R^2^) for each fitting equation exceeded 0.99, indicating a strong linear relationship between the voltage output and torque input at room temperature, which aligns with the theoretical output characteristics of the Wheatstone full-bridge circuit.

## 5. Research on Temperature Compensation Algorithm

**Figure 13:**
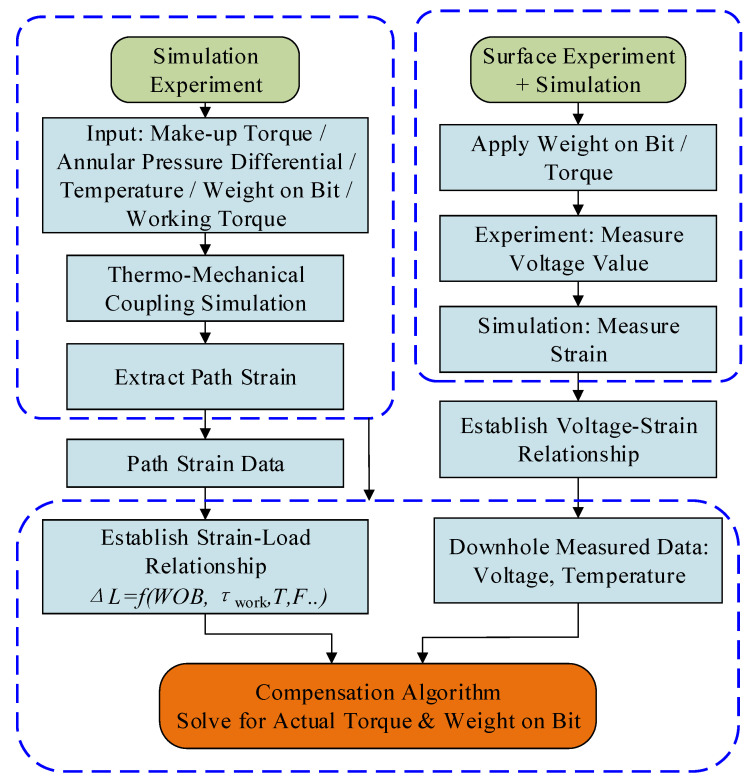
Development of the temperature compensation algorithm.

Based on structural strain data acquired from thermo-mechanical coupling simulations and combined with experimental fitting results, a temperature-compensation algorithm using polynomial regression was developed in this study, with its implementation workflow illustrated in Figure 13. The core concept of the algorithm involves establishing a mapping relationship among strain, temperature, and load to eliminate the temperature-induced effects on strain measurements. By utilizing measured temperature and voltage values, the algorithm enables the inverse deduction of the actual WOB and torque values.

### 5.1. Polynomial Regression Model

The algorithm employs a polynomial regression model that comprehensively accounts for the effects of temperature on both zero-point drift and sensitivity variations. Independent bivariate quadratic polynomial regression models were developed for WT (*τ*_work_) and WOB, respectively. For torque measurement, the input variables are the measured temperature (*T*) and the XY-direction shear microstrain (*με*_xy_), while the output variable is the WT.

To address the dual effects of temperature on sensor signals—namely, zero-point drift and sensitivity variation—a bivariate quadratic polynomial form was adopted for the compensation model. This form captures not only the individual linear influences of temperature and strain but also their interactive effects and nonlinear behaviors. Separate models were developed for Working Torque and WOB. For torque, the model predicts WT (*τ*_work_) from measured temperature (*T*) and XY-direction shear microstrain (*με*_xy_):

(29) $$\tau_{work}=\gamma_{0}+\gamma_{1}T+\gamma_{2}\mu \varepsilon_{\mathrm{xy}}+\gamma_{3}T^{2}+\gamma_{4}T\mu \varepsilon_{\mathrm{xy}}+\gamma_{5}\mu \varepsilon_{\mathrm{xy}}^{2}$$

Similarly, for WOB measurement, the input variables are the measured temperature (*T*) and the X-direction normal microstrain (*με*_x_), while the output variable is the working WOB:

(30) $$F_{W}=\beta_{0}+\beta_{1}T+\beta_{2}\mu \varepsilon_{x}+\beta_{3}T^{2}+\beta_{4}T\mu \varepsilon_{x}+\beta_{5}\mu \varepsilon_{x}^{2}$$

The polynomial coefficients were solved using the least-squares method based on the existing 240 sets of simulation data containing combinations of temperature, WOB, and torque, with the coefficient matrix denoted as X. During the solution process, L2 regularization was introduced. Regularization constrains the magnitude of the function by adding a penalty term to the loss function [36,37]. The optimized objective function is expressed as:

(31) $$\min_{\theta }\left\{{\left\|Y-X\theta \right\|}_{2}^{2}+\lambda {\left\|\theta \right\|}_{2}^{2}\right\}$$

In Equation (31), **X** is the design matrix containing the measured temperature and strain values (including quadratic and interaction terms), **Y** is the vector of true load values (Working Torque or WOB), and **θ** is the vector of unknown regression coefficients to be estimated. The forward model is given by Equations (29) and (30), which are quadratic polynomials in temperature and strain. The inversion aims to find *θ* that minimizes the regularized residual sum of squares. Here, **X** ∈ R*^n^*^×6^, **Y** ∈ R*^n^*, and **θ** ∈ R^6^ for the quadratic model, $∥Y-X\theta {∥}_{2}^{2}$ is the residual sum of squares, λ is the regularization parameter controlling penalty strength, and $∥\theta {∥}_{2}^{2}$ is the L2 penalty on the coefficient magnitudes. The regularization parameter *λ* controls the penalty strength on the coefficient magnitudes. It was determined via five-fold cross-validation across 240 simulation datasets, with the value minimizing the average validation error chosen. This ensures the model generalizes well to unseen data. The analytical solution that minimizes this objective function is:

(32) $$\theta ={\left(X^{⊤}X+\lambda I\right)}^{-1}X^{⊤}y$$

Applying this solution to the simulation data yielded the final, robust coefficients presented in Table 5.

The coefficients in Table 5 reveal the model’s key characteristics. For both models, the dominant linear strain coefficients (*γ*_2_, *β*_2_) confirm that the sensor’s primary response is to mechanical load, as designed. The presence of significant temperature-strain interaction coefficients (*γ*_4_, *β*_4_) quantifies the core thermal interference effect that the compensation must correct. Their opposite signs for torque and WOB models reflect the different physical nature of thermo-mechanical coupling under torsion versus axial compression. The inclusion of quadratic terms (*γ*_3_, *γ*_5_, *β*_3_, *β*_5_) allows the model to capture nonlinear drifts. Applying L2 regularization ensures these coefficients yield a robust model, preventing overfitting to the finite simulation data and enhancing reliability for field use.

### 5.2. Algorithm Validation

To evaluate the performance of the temperature compensation algorithm, an independent validation experiment was designed. The validation dataset comprises 45 sets of measured data obtained from independent calibration experiments using the setup described in Section 4.1 (Figure 12), covering temperature ranges of 80–140 °C and operational condition combinations of 0–200 kN WOB and 0–10 kN·m torque.

**Figure 14:**
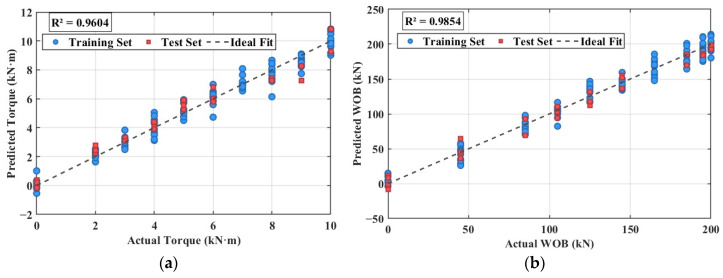
Regression model prediction results (**a**) Torque prediction (**b**) WOB prediction.

The predictive performance of the compensation models is evaluated against the independent test set, with the results visualized in Figure 14. Figure 14a presents the results for the torque model. The predicted torque values closely match the actual values, evidenced by a high coefficient of determination (R^2^) of 0.9604. The data points are distributed tightly along the ideal 1:1 reference line. For the WOB model, Figure 14b shows a similarly strong correlation. The data points cluster closely around the reference line. The R^2^ value reaches 0.9854, meaning the model explains 98.54% of the variance in the test data, confirming its satisfactory predictive capability.

**Figure 15:**
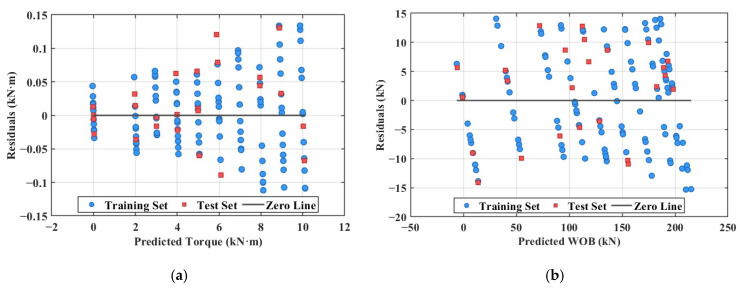
Residual distribution (**a**) Torque residual distribution (**b**) Weight on bit residual distribution.

Figure 15 presents the residual distribution of the models, which further reveals the error characteristics of the algorithm. As shown in Figure 15a, the residuals of the torque model exhibit desirable random characteristics, with most residuals confined within ±0.8 kN·m. The mean absolute error (MAE) is 0.49 kN·m, corresponding to a relative error of 4.90%. Figure 15b demonstrates that the residuals of the WOB model are randomly distributed around the zero line, with no apparent patterns or trends. The maximum absolute residual is 15.2 kN, with an MAE of 8.15 kN and a relative error of 4.07%. The uniform distribution of residuals indicates a reasonable model specification, with no systematic prediction bias.

Furthermore, no significant heteroscedasticity is observed in the residual distribution, confirming that the residual variance remains relatively stable across different predicted value levels.

Validation results based on the test set demonstrate that all performance metrics of the developed temperature compensation algorithm meet industrial standards for MWD systems. Furthermore, since the inversion is based on an analytical solution (Equation (32)), the compensation algorithm requires no iterations and involves only a few floating-point operations per measurement point, making it suitable for real-time implementation on downhole embedded systems. Both WOB and torque measurements achieve relative errors within 5%, while the algorithm’s stable performance across the entire temperature range establishes a theoretical foundation for its engineering application in high-temperature environments of deep and ultra-deep wells [38].

## 6. Conclusions

This study addresses thermal-induced measurement errors in bit-parameter recorders under high-temperature deep-well conditions by proposing a temperature-compensation method based on thermo-mechanical coupling simulation and polynomial regression. The equations presented in this paper serve distinct roles within our methodology: Equations (1)–(10) establish the fundamental operating principles of the strain gauge sensors and their relationship to mechanical loads (WOB and torque). Equations (11)–(17) quantify the thermal influence mechanisms on both material properties and sensor circuitry, forming the theoretical basis for temperature-induced errors. Equations (18)–(23) define the thermo-mechanical coupling simulation framework, enabling quantitative mapping between temperature, loads, and structural strain. Finally, Equations (24)–(30) underpin the inversion-based compensation algorithm, with Equations (29) and (30) serving as the core bivariate quadratic regression models that directly compute compensated WOB and torque values from measured temperature and voltage inputs. This integrated equation framework systematically bridges physical understanding, simulation modeling, and practical compensation implementation. The following conclusions are drawn through simulation and experimental validation:

(1) The integration of physical simulation and data-driven approaches effectively resolves the complex nonlinear challenges in temperature compensation. The thermo-mechanical coupling simulation accurately captures the physical mapping relationships among temperature, strain, and load. Based on this foundation, the developed bivariate quadratic polynomial regression model successfully maps the complex coupling between variables, demonstrating the stable performance of the temperature compensation algorithm across the entire operating temperature range.
(2) The proposed temperature-compensation algorithm significantly enhances the measurement accuracy of MWD systems in high-temperature environments. The quadratic polynomial regression model, established using thermo-mechanical coupling simulation data, exhibits excellent performance on the test set: torque measurement achieves a mean absolute error of 0.49 kN·m (relative error 4.90%) with a coefficient of determination R^2^ of 0.9604; WOB measurement shows a mean absolute error of 8.15 kN (relative error 4.07%) with R^2^ reaching 0.9854. This accuracy level substantially surpasses the typical 10–20% error range of conventional uncompensated methods in high-temperature environments.

The temperature compensation scheme demonstrates promising potential for engineering applications. The algorithm requires only conventional measurement signals such as temperature sensor readings and strain gauge voltages as inputs, features low computational complexity, and maintains the original structural configuration, making it suitable for limited downhole space. These findings provide a reliable solution for temperature-induced errors in MWD systems for deep and ultra-deep wells, contributing significantly to the advancement of high-temperature, high-pressure drilling technology. Future research could enhance the adaptive capability and long-term robustness of the compensation framework under unexpected drilling dynamic conditions by incorporating online learning mechanisms. Furthermore, extending this methodological framework to temperature drift correction for broader MWD parameters (such as near-bit vibration and annular pressure) represents a valuable direction for subsequent investigation.

## Figures and Tables

**Table 1:** Mesh information.

Model Type	Model I	Model II	Model III	Model IV	Model V
**Amount of mesh**	319,925	406,479	523,918	625,178	718,226

**Table 2:** Torque Calibration Experimental Results.

Number	WT/kN·m	Voltage 1 /mv	Voltage 2 /mv	Voltage 3 /mv	Average Voltage /mv	XY Shear Microstrain (με_xy_)
1	0	1202.10	1201.40	1201.70	1201.73	0
2	1.0	1236.30	1235.80	1235.80	1235.97	455.66
3	2.0	1271.20	1271.10	1270.20	1270.83	472.79
4	3.0	1305.20	1305.30	1305.20	1305.23	488.14
4	4.0	1339.90	1340.40	1340.40	1340.23	495.77
6	5.0	1375.30	1375.30	1375.10	1375.23	503.40
7	6.0	1408.80	1409.90	1409.30	1409.33	511.01
8	7.0	1443.20	1443.00	1443.20	1443.13	518.64
9	8.0	1477.10	1477.10	1476.40	1476.87	526.27
10	9.0	1511.20	1510.70	1511.40	1511.10	530.02
11	10.0	1545.70	1545.90	1545.90	1545.83	531.85

**Table 3:** WOB Calibration Experimental Results.

Number	WOB/kN	Voltage 1 /mv	Voltage 2 /mv	Voltage 3 /mv	Average Voltage /mv	X Normal Microstrain (με_x_)
1	0	1326.20	1326.30	1326.50	1326.33	0
2	20	1337.30	1338.10	1338.30	1337.90	6.58
3	40	1348.60	1348.70	1349.30	1348.87	7.56
4	60	1360.40	1360.80	1361.20	1360.80	9.21
4	80	1371.30	1371.40	1371.60	1371.43	10.40
6	100	1382.60	1382.40	1382.60	1382.53	11.65
7	120	1393.80	1393.60	1393.80	1393.73	12.88
8	140	1404.60	1404.40	1404.20	1404.40	14.59
9	160	1415.20	1415.50	1415.00	1415.23	15.36
10	180	1426.20	1425.90	1425.40	1425.83	16.68
11	200	1436.20	1436.30	1436.30	1436.27	18.30

**Table 4:** Fitting Coefficients and Evaluation Metrics.

Model	K	B	R^2^	RMSE	MAPE
WT-Voltage	0.0291	−34.956	0.99	0.025 kN·m	0.391%
WOB-Voltage	1.815	−2407.1	0.99	0.607 kN	0.592%
Voltage-με_x_	0.6467	1201.73	0.99	1.132 mv	0.067%
Voltage-με_xy_	6.0332	1326.33	0.99	0.998 mv	0.056%

**Table 5:** Parameters of the polynomial regression model.

	$\gamma_{0}$ $,\;\beta_{0}$	$\gamma_{1}$ $,\;\beta_{1}$	$\gamma_{2}$ $,\;\beta_{2}$	$\gamma_{3}$ $,\;\beta_{3}$	$\gamma_{4}$ $,\;\beta_{4}$	$\gamma_{5}$ $,\;\beta_{5}$
Torque Regression	−2.09	−0.043	−0.0741	1.45 × 10^−4^	1.44 × 10^−4^	6.4 × 10^−5^
WOB Regression	−476.59	−0.81	0.11	−3.32 × 10^−3^	−3.13 × 10^−3^	2.45 × 10^−3^

## Data Availability

The original contributions presented in this study are included in the article. Further inquiries can be directed to the corresponding author.
